# Individual or mixing extrusion of Tartary buckwheat and adzuki bean: Effect on quality properties and starch digestibility of instant powder

**DOI:** 10.3389/fnut.2023.1113327

**Published:** 2023-03-21

**Authors:** Zhuo Zhang, Yongqiang Liang, Liang Zou, Yunan Xu, Mengzhuo Li, Bao Xing, Manli Zhu, Yichen Hu, Guixing Ren, Lizhen Zhang, Peiyou Qin

**Affiliations:** ^1^Key Laboratory of Chemical Biology and Molecular Engineering of Ministry of Education, School of Life Science, Shanxi University, Taiyuan, China; ^2^Key Laboratory of Quality Evaluation and Nutrition Health of Agro-Products, Ministry of Agriculture and Rural Affairs, Institute of Crop Sciences, Chinese Academy of Agricultural Sciences, Beijing, China; ^3^Key Laboratory of Coarse Cereal Processing, Ministry of Agriculture and Rural Affairs, Sichuan Engineering and Technology Research Center of Coarse Cereal Industrialization, School of Food and Biological Engineering, Chengdu University, Chengdu, China; ^4^Seed Administration Station of Shijiazhuang, Shijiazhuang, China

**Keywords:** instant powder, Tartary buckwheat, adzuki bean, extrusion, flavonoids, α-glucosidase inhibitory activity, *in vitro* starch digestibility

## Abstract

**Introduction:**

Tartary buckwheat and adzuki bean, which are classified as coarse grain, has attracted increasing attention as potential functional ingredient or food source because of their high levels of bioactive components and various health benefits.

**Methods:**

This work investigated the effect of two different extrusion modes including individual extrusion and mixing extrusion on the phytochemical compositions, physicochemical properties and *in vitro* starch digestibility of instant powder which consists mainly of Tartary buckwheat and adzuki bean flour.

**Results:**

Compared to mixing extrusion, instant powder obtained with individual extrusion retained higher levels of protein, resistant starch, polyphenols, flavonoids and lower gelatinization degree and estimated glycemic index. The α-glucosidase inhibitory activity (35.45%) of the instant powder obtained with individual extrusion was stronger than that obtained with mixing extrusion (26.58%). Lower levels of digestibility (39.65%) and slower digestion rate coefficient (0.25 min^−1^) were observed in the instant powder obtained with individual extrusion than in mixing extrusion (50.40%, 0.40 min^−1^) by logarithm-of-slope analysis. Moreover, two extrusion modes had no significant impact on the sensory quality of instant powder. Correlation analysis showed that the flavonoids were significantly correlated with physicochemical properties and starch digestibility of the instant powder.

**Discussion:**

These findings suggest that the instant powder obtained with individual extrusion could be used as an ideal functional food resource with anti-diabetic potential.

## 1. Introduction

Instant powder is one of the most popular baked foods worldwide, which includes various advantages, such as long shelf life, convenient packaging, and extensive consumption. As awareness increases about consuming high-quality foods, it becomes necessary to seek cereal flour with better edible and health care value to produce instant powder. Cardiometabolic diseases such as diabetes and cardiovascular disease have caused a huge global health burden, at the same time, coarse grains consumption has been considered as a major factor in controlling heart metabolic diseases (1). Coarse grains include grain foods (e.g., adlay, buckwheat, and adzuki bean) other than wheat and rice and are similar to whole grains, have received widespread attention for their prominent potential health benefits (2, 3).

Tartary buckwheat [*Fagopyrum tataricum* (L.) Gaench], which is a species of buckwheat, has attracted increasing attention from food scientists for its health effects over chronic diseases (4). Tartary buckwheat grains contain a variety of nutrients, mainly including protein, polysaccharide, starch, lipid, rutin, polyphenols and elements (5). The unique composition of Tartary buckwheat contributes to diverse health benefits such as antioxidant, antitumour, hypotensive, hypoglycaemic, and hypolipidaemic activities (6). Moreover, epidemiological studies showed that people who use Tartary buckwheat as the main food have a rather low occurrence of chronic diseases such as diabetes and hypertension (7). Various parts of Tartary buckwheat have been used in traditional medicine to treat a series of stubborn and chronic diseases (6). At present, Tartary buckwheat has been processed into various foods in China, such as tea, alcoholic beverages, vinegar, noodles, porridge, biscuits, cakes and bean sprouts. Adzuki bean (*Vigna angularis*) is a kind of edible bean, which is one of the earliest crops in East Asia (8). Adzuki bean has been receiving increasing attention as a potential functional ingredient or food that is rich in a range of nutrients including bioactive carbohydrates, protein, flavonoids and saponins. These nutritious substances endow adzuki bean with various health benefits such as anti-inflammatory, anti-cancer, anti-tumor, anti-diabetes and anti-hypertension properties (9, 10). Liu et al. (9) reported that flavonoids in adzuki bean could inhibit α-glucosidase activity to achieve anti-diabetes effect.

However, the nutritional value of Tartary buckwheat and adzuki bean are not always well understood and appreciated by consumers. These problems could be solved by processing new food with extrusion technology (11). Extrusion is one of the widely used technologies for producing puffed food, which uses high temperatures, pressures and shear forces to change the structure of a material and can be used to produce a variety of breakfast cereals and snacks from cereal grains (12). Extrusion technology has also utilized pseudocereals for manufacturing nutritious products owing to the interest of the people in functional foods. Specially, many researchers have evaluated pseudocereals and their blends in extrusion process (13). Individual extrusion is to extrude a specific grain and then mix it with other ingredients. Chang et al. (14) found that the whole grain barley showed lower lightness values and higher gelatinization degree after extrusion. Our previous research also indicated that extrusion can improve the gelatinization degree and digestibility of Tartary buckwheat flour (15). On the other hand, mixing extrusion is to extrude two or more kinds of grain mixed flour. Singh et al. (13) reported that the corn flour containing buckwheat showed stronger redness and lower water absorption index (WAI) after extrusion. The corn-based extrudates containing amaranth/quinoa/kañiwa presented the higher sectional expansion index than pure corn extrudates (16). However, the effects of individual extrusion and mixing extrusion on the physicochemical properties and starch digestibility of cereal products have not been compared.

We assumed that different extrusion treatments have different influences on the quality properties and starch digestibility of instant powder. Therefore, two processing modes were performed in this study to investigate their influence on phytochemical composition and properties, α-glucosidase inhibitory activity, sensory properties and *in vitro* starch digestibility of instant powder: One is called individual extrusion (IE), which is to extrude Tartary buckwheat flour and adzuki bean flour individual, and then mix together with other ingredients (walnut, wolfberry, xylitol). The other is called mixing extrusion (ME), which is to mix Tartary buckwheat flour and adzuki bean flour before extruding, and then mix with other ingredients (walnut, wolfberry, xylitol). This study will provide theoretical basis for further development of instant coarse grain food and improvement of processing technology.

## 2. Materials and methods

### 2.1. Materials

Tartary buckwheat, adzuki bean, walnut, wolfberry and xylitol were kindly provided by Ningxia Huantai Bio-Technology Co., Ltd (Ningxia, China). Standard reagents (gallic acid, rutin, quercetin, myricetin, vitexin and isovitexin), α-glucosidase (100 U) and p-nitrophenyl-a-D-glucopyranoside (pNPG) were purchased from Shanghai Yuanye Bio-Technology Co., Ltd (Shanghai, China). Pepsin (Sigma P7000, from porcine gastric mucosa) and pancreatin enzyme (Sigma P7545, from porcine pancreas) were purchased from Sigma Aldrich Co (St. Louis, MO, United States). The other chemicals and reagents used in this study were of analytical grade.

### 2.2. Processing procedures

**Figure 1:**
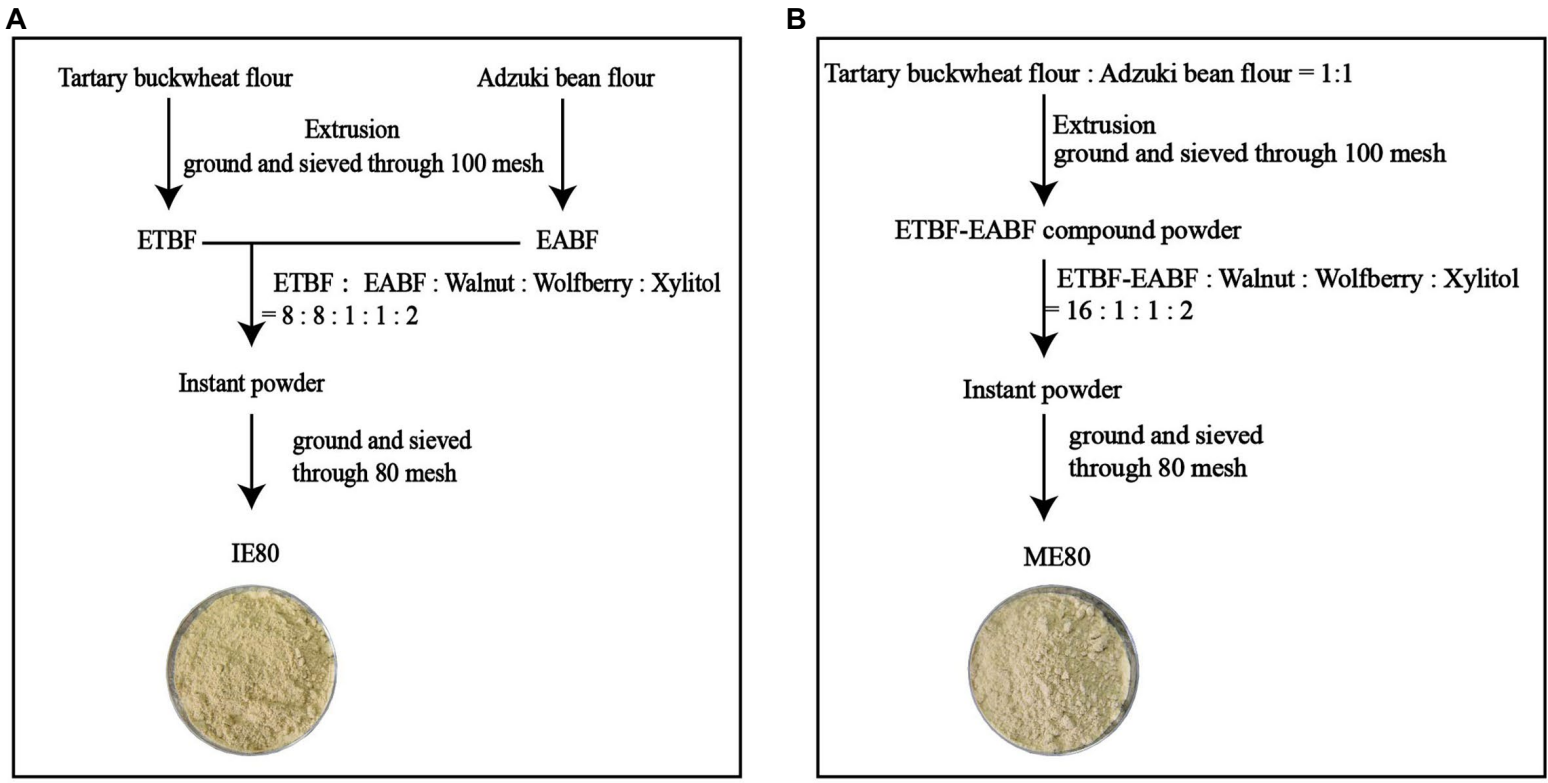
Simplified flow diagram of instant powder preparation procedures. **(A)** individual extrusion; **(B)** mixing extrusion. ETBF, extruded Tartary buckwheat flour; EABF, extruded adzuki bean flour. ETBF-EABF, mixed extrudates of Tartary buckwheat flour and adzuki bean flour; IE80, instant powder obtained with individual extrusion, 80 mesh; ME80, instant powder obtained with mixing extrusion, 80 mesh.

The detailed processing procedures are shown in Figure 1. Extrusion operations were carried out using a twin screw extruder (DXY-85, Jinan, China). For extrusion parameters, the extrusion temperature was 120 ^o^ C, the feed moisture content (dry basis) was 14%, the feeding rate was 120 kg/h, and the screw speed 590 rpm. Figure 1A represents instant powder obtained with IE and Figure 1B represents instant powder obtained with ME. The ratio of instant powder was adjusted to Tartary buckwheat flour: 40%; adzuki bean flour: 40%; walnut: 5%; wolfberry: 5%; xylitol: 10%. The obtained instant powder was ground and passed through 80 mesh sieves, which are, respectively, recorded as IE80 and ME80.

### 2.3. Nutritional composition analysis

The compositions of different instant powder samples including moisture, protein, fat and ash were determined according to the methods of GB5009.5–2016, 5009.6–2016, 5009.4–2016. The total starch content was determined by kit assays (Megazyme International Ireland Ltd., Wicklow, Ireland).

### 2.4. Analysis of total polyphenols, total flavonoids and individual flavonoid compounds

The total polyphenols, total flavonoids and individual flavonoid compounds were extracted and measured following the method of Qin et al. (17) with slight modifications. In brief, the total phenolic contents of extracts were determined according to Folin–Ciocalteus method and expressed as micrograms of gallic acid equivalent per gram of sample, total flavonoids content was determined using the aluminium chloride colorimetric method and expressed as micrograms of rutin equivalent per gram of sample.

After the samples were passed through a 0.45 μm PEC syringe filter membrane (Jinteng, Tianjin, China), the individual flavonoid compounds including rutin and quercetin were analyzed by an Alltech HPLC system (Alltech, Chicago, United States). The analytical column was a PerkinElmer® column (250 mm × 4.6 mm, Sheiton, United States) and the wavelength of the UV detector was set at 375 nm. The mobile phase was 0.05% trifluoroacetic acid aqueous solution (A) and 100% acetonitrile (B). A gradient flow system was established as follows with a flow rate of 1 mL/min: 0–5 min, 15–20% B; 5–25 min, 20–25% B; 25–35 min, 25–100% B; 35–40 min,100% B; 40–43 min, 100–15% B; and 43–48 min, 15% B. The column temperature was kept at 30 ^o^ C and the injection volume was 20 μL.

### 2.5. Color determination

The color parameters of different instant powder samples were determined according to a procedure described by our laboratory (5) with slight modifications. The colorimeter was calibrated using a standard white plate. Thirty replicate measurements were performed before the color parameters were recorded. Whiteness index (WI) was calculated based on the following Eq. 1:


(1) $$\mathrm{WI}=100-\sqrt{{\left(100-L*\right)}^{2}+a{*}^{2}+b{*}^{2}}$$

Where L* means lightness (0 for black and 100 for white), a* is red (+) to green (−), and b* is yellow (+) to blue (−).

### 2.6. Degree of gelatinization analysis

The DG of different instant powder samples were determined following a previous method (15). The sample (50 mg) was dispersed in 50 mL of 0.05 mol/l KOH solution and continuously shaken for 20 min. The slurry was then centrifuged at 3000 g for 10 min, and the supernatant (1 mL) and 0.05 M HCl were mixed in equal proportions and diluted with distilled water to a volume of 10 mL. Subsequently, 0.1 mL of iodine solution was added and mixed well. The absorbance of the mixture was measured at 600 nm. In the above steps, KOH (0.05 M) and HCl (0.05 M) were replaced with KOH (0.5 M) and HCL (0.5 M) as sample controls. The DG value was calculated according to the following Eq. 2:


(2) $$DG=A_{1}/A_{2}\times 100\%$$

Where A_1_ and A_2_ represent the absorbance of the test sample and the control, respectively.

### 2.7. Hydration characteristics analysis

The water absorption index (WAI), water solubility index (WSI), and swelling power (SP) of instant powder were determined according to a previous procedure (15) with small modifications. In brief, 2 g of the sample was dispersed in 25 mL of distilled water and incubated in a water bath at 30 ^o^ C for 30 min with stirring every 10 min. The slurry was then centrifuged at 3100 g for 15 min, and the supernatant was transferred to an aluminum container. Subsequently, the supernatant and sediment were, respectively, dried to constant weight at 105 ° C. The WAI, WSI and SP were calculated according to the following Eqs 3–5:


(3) $$\mathrm{WAI}\;\left(g/g\right)=\frac{W_{1}}{W_{0}}$$

(4) $$\mathrm{WSI}\;\left(g/g\right)=\frac{W_{2}}{W_{0}}$$

(5) $$\mathrm{SP}=\frac{W_{1}}{W_{0}\times \left(1-\mathrm{WSI}\right)}$$

Where W_0_ = the weight of the instant powder × (1–moisture content (%)). W_1_ is the weight of the sediment, and W_2_ is the weight of the solid dissolved in the supernatant.

### 2.8. α-glucosidase inhibitory activity

The instant powder samples (0.5 g) were accurately weighed and mixed with 30 mL of 70% (v/v) methanol solution. Each suspension was incubated in a water bath at 65 ^o^ C for 2 h and then filtered. The supernatants were evaporated to dryness by vacuum rotary evaporator, then resolved with 70% ethanol solution and used for subsequent analysis.

The α-glucosidase inhibitory activity of flavonoid extracts was assessed by using previous method (18) with slight modifications. The sample solution (50 μL) was mixed with 120 μL of the 0.5 U/mL α-glucosidase solution. After incubation at 37 ^o^ C for 10 min, 120 μL 2.5 mM pNPG in phosphate buffer solution was added. Then the mixture was incubated at 37^o^C for 15 min and the reaction was terminated by adding 480 μL 0.2 M sodium carbonate. Finally, the absorbance was measured at λ = 405 nm. Acarbose was used as the positive control. The α-glucosidase inhibitory activity was calculated using the following formula:


(6) $$\mathrm{Inhibition}\;\left(\%\right)=\left(A_{0}–A_{1}\right)/A_{0}\times 100$$

Where A_0_ and A_1_ represent the absorbance of the control and the experimental samples, respectively. The α-glucosidase inhibitory ability of the extracts was characterized by inhibiting 50% of the enzymatic activity (IC_50_).

### 2.9. Sensory evaluation

Sensory evaluation of instant powder was conducted according to Chen et al. (19) with slight modification. As shown in Table 1, the sensory evaluation criteria are taste, aroma, color, tissue state, and soakage. A group of 8 trained panelists (aged 20–30 years) from Institute of Crop Sciences, Chinese Academy of Agricultural Sciences participated in the sensory analyses. Overall acceptability score was calculated by averaging of whole sensory parameters.

**Table 1:** The sensory evaluation criteria of different instant powder samples.

Items	The grading standards	Scores
Taste	The taste is fine and smooth, the consistency is moderate, no grain feeling, has the appropriate sweet without bitter taste	21–30
	The taste is fine and smooth, the consistency is moderate, fine granules, moderate sweetness and slight bitterness	11–20
	The taste is rough, the consistency is thin, too sweet or bitter	0–9
Aroma	It has the aroma of Tartary buckwheat, red beans and walnuts, but none of them is particularly outstanding	15–20
	A certain fragrance is too strong, and there is no other peculiar smell	7–14
	The smell is not correct, there are other peculiar smells	0–6
Color	Brownish yellow, uniform distribution	15–20
	Brownish yellow and brownish, relatively uniform distribution	7–14
	Brownness, maldistribution	0–6
Tissue state	The powder is loose without caking	11–15
	The powder is relatively loose with a little caking	6–10
	The powder is rough with a lot of caking	0–5
Soakage	Quick wetting, no caking and stratification	11–15
	The wetting is slow, with a small amount of caking and slight stratification	6–10
	Slow wetting, more caking and obvious stratification	0–5

### 2.10. *In vitro* starch digestibility and estimated glycemic index

The digestion of instant powder was performed according to a previous method (15). Briefly, 100 mg of sample was dispersed in 6 mL of distilled water, followed by the addition of 5.0 mL pepsin solution (440 U/mL in 0.02 M HCl). After incubation at 37 ^o^ C for 30 min, 5.0 mL of 0.02 M acetate buffer and 8 mL of enzyme solution (2 mg of pancreatin enzyme plus 100 mL 3,260 U/mL amyloglucosidase in 7.9 mL of 0.02 M acetate buffer, pH 6.0) were added. The reaction solution was incubated at 37.0 ^o^ C with moderate stirring at 50 rpm. Aliquots were taken at 0, 5, 10, 15, 20, 30, 45, 60, 90, 120, 150, and 180 min to determine the glucose released by the D-glucose (GOPOD format) assay, which was then transformed into the amount of digested starch molecules.

According to previous study reported by Englyst et al. (20), three different starch fractions are defined as follows: Rapidly digestible starch (RDS): amount of glucose release after 20 min; Slowly digestible starch (SDS): amount of glucose released between 20 and 120 min of *in vitro* digestion; Resistant starch (RS): total starch minus amount of glucose released within 120 min of *in vitro* digestion. The contents of RDS, SDS and RS were calculated according to the following Eqs 7–9:


(7) $$\mathrm{RDS}\;\left(\%\right)=\left(G_{20}–G_{0}\right)\times 0.9/\mathrm{TS}\times 100$$

(8) $$\mathrm{SDS}\;\left(\%\right)=\left(G_{120}–G_{20}\right)\times 0.9/\mathrm{TS}\times 100$$

(9) $$\mathrm{RS}\;\left(\%\right)=100–\mathrm{RDS}\;\left(\%\right)–\mathrm{SDS}\;\left(\%\right)$$

Where G_0_, G_20_, G_120_ are the glucose released (mg) within 0, 20, and 120 min, respectively. TS is weight of total starch (mg) in the sample. 0.9 (162/180) is the factor to convert from free D-glucose to anhydro-D-glucose as occurs in starch.

The hydrolysis index (HI) was calculated as the area under the starch hydrolysis curves (AUC) of a sample as a percentage of the corresponding AUC of glucose (21). The estimated glycemic index (eGI) was estimated using Eq. 10:


(10) eGI=(0.549×HI)+39.71

### 2.11. Fitting to first-order kinetics

The starch digestion curves were fitted to an integrated first-order Eq. 11:


(11) $$C_{t}=\left(C_{\infty }-C_{0}\right)\times \left(1-e^{-\mathrm{kt}}\right)+C_{0}$$

Where C_t_ and C_0_ are the starch digestion ratios at time t and 0 (min), respectively; C_∞_ is the starch hydrolysis rate at the end of the digestion; and k is the starch digestion rate coefficient.

Different digestion phases were identified by using the logarithm-of-slope (LOS) analysis method described by Zou et al. (22) through a transformed Eq. 12:


(12) $$\ln\;\frac{{\mathrm{dC}}_{t}}{\mathrm{dt}}=\ln\;\left(C_{\infty }-C_{0}\right)-\mathrm{kt}$$

Thus, a plot of ln (dC/dt) against time has a slope k and intercept ln (C_∞_–C_0_), from which C_∞_ can be obtained. The slope in this study could be estimated from the fraction (C_2_–C_1_)/ (t_2_–t_1_), (C_3_–C_2_)/ (t_3_–t_2_) etc. And the natural logarithms plotted against the relevant, mean time, i.e., (t_2_ + t_1_)/2, (t_2_ + t_3_)/2 etc. A spread sheet can be set up to perform these relatively simple calculations. For substrates containing starch fractions digested at a single rate, the LOS plot is linear, while others may have multiple distinct linear phases. Therefore, the whole starch digestion can be expressed by a piecewise function. In the current study, we found that the plot is nonlinear, with two different linear regions with different slopes. C_0_ is determined experimentally, while the rest three parameters fast digestion rate coefficient (k_f_), slow digestion rate coefficient (k_s_) and C_∞_ are determined by the non-linear least-squares refinement in Excel to the global minimum. A complete fitting process was reported by Li et al. (23).

### 2.12. Statistical analysis

Experimental results were expressed as mean ± standard deviation, and three independent experiments except color parameters (30 replicate) were performed. Statistical analysis was performed using SPSS version 26.0 (SPSS Inc., Chicago, IL, United States). The data were subjected to one-way analysis of variance (ANOVA) to determine the differences between samples. Significant differences were compared by Duncan test on the level of *p* < 0.05. The correlation matrix analysis was analyzed with the Pearson correlation coefficient.

## 3. Results and discussion

### 3.1. Phytochemical compositions

As shown in Table 2, the instant powder obtained with IE contained significant higher amounts of protein (15.88%), ash (2.51%) and lower amount of starch (50.08%), fat (4.00%) in comparison with instant powder obtained with IE (13.03, 1.86, 56.62 and 4.69%, respectively; *p* < 0.05). Previous studies have proved that extrusion treatment can significantly decrease the content of starch, protein and fat (24, 25). Therefore, our results indicated that the instant powder obtained with IE had less loss of protein and ash, whereas instant powder obtained with ME had less loss of starch and fat.

**Table 2:** Phytochemical compositions of different instant powder samples.

Materials	Moisture*	Starch*	Protein*	Fat*	Ash*	TP**	TF**	Rutin**	Quercetin**
IE80	6.03 ± 0.08b	50.08 ± 0.58b	15.88 ± 0.08a	4.00 ± 0.05b	2.51 ± 0.02a	5.09 ± 0.05a	6.37 ± 0.04a	5.69 ± 0.32a	0.09 ± 0.01a
ME80	6.57 ± 0.15a	56.62 ± 0.34a	13.03 ± 0.05b	4.69 ± 0.09a	1.86 ± 0.02b	2.67 ± 0.02b	2.06 ± 0.09b	1.48 ± 0.09b	0.04 ± 0.00b

Different letters in the same column indicate significant differences (*p* < 0.05). TP, total polyphenols; TF, total flavonoids; IE80, instant powder obtained with individual extrusion, 80 mesh; ME80, instant powder obtained with mixing extrusion, 80 mesh.

* Expressed as % of dry weight.

** Expressed as mg/g of dry weight.

**Figure 2:**
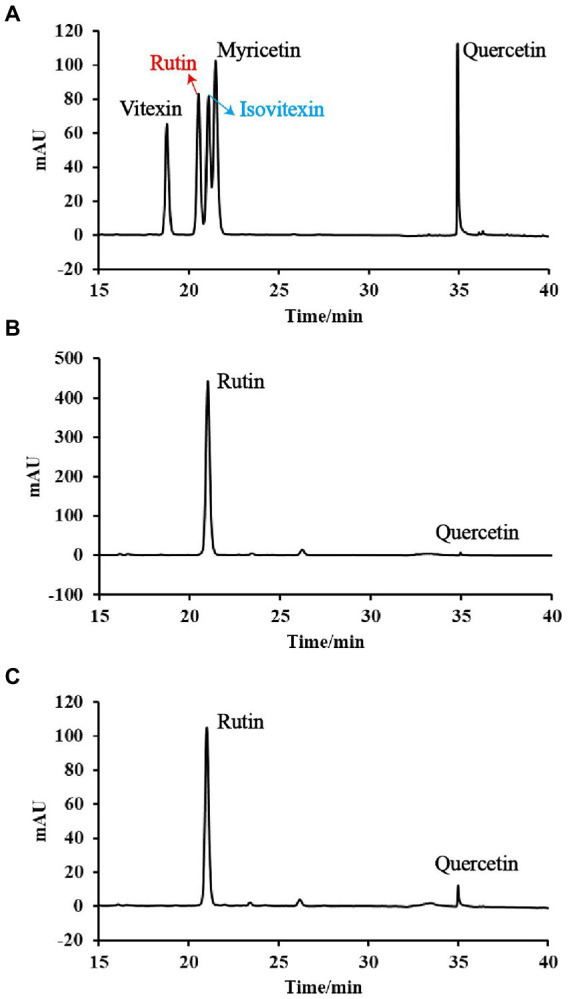
HPLC profiles of different instant powder samples: **(A)** standards; **(B)** instant powder obtained with individual extrusion; and **(C)** instant powder obtained with mixing extrusion.

The contents of total polyphenols, total flavonoids and individual flavonoid compounds are presented in Table 2. The instant powder obtained with IE showed higher content of total polyphenols (5.09 mg/g) and total flavonoids (6.37 mg/g), rutin (5.69 mg/g) and quercetin (0.09 mg/g) than ME (2.67, 2.06, 1.48, 0.04 mg/g, *p* < 0.05). The majority of flavonoids in instant powder were rutin, accounting for 71.84% (IE80) and 89.32% (ME80), and the proportion of quercetin was 1.41% (IE80) and 1.94% (ME80). Besides, as shown in Figure 2, the specific flavonoids (such as myricetin, vitexin and isovitexin) both in IE80 and ME80 did not reach the detection limit (data was not shown in Table 2). This suggested that phenolic compounds were greatly lost during extrusion. Korus et al. (26) also observed that dry beans (*Phaseolus vulgaris* L.) lost 80% of its myristin content and 50% of its quercetin concentration during extrusion. Wang et al. (27) revealed that rutin and quercetin in flavonoids interacted with starch through non-covalent bond to form a complex. Moreover, rutin in flavonoids can also combine with soy protein isolates to form complex through hydrophobic interaction (28). Therefore, perhaps the reason that the more loss of flavonoids in ME80 compared to IE80 is that the flavonoids in Tartary buckwheat interact with the macromolecular components in adzuki bean.

### 3.2. Color attributes

Color is one of the key quality parameters that directly affect consumer acceptance. Color characteristics reflect starch gelatinization, caramelization, and Maillard reaction induced by extrusion (29). Previous studies have shown that extrusion could lead to Maillard reactions and a reduction of the lipids oxidation due to enzymes inactivation that induces the formation of melanoidins and the pigments protection, which in turn produces the modification of the flours color (30, 31). The color parameters (L*, a*, b*, WI) are shown in Table 3. The instant powder obtained with ME showed higher lightness (67.95) and WI values (58.65) on average compared to instant powder obtained with IE (65.88, 54.93, *p* < 0.05). In contrast, instant powder obtained with IE showed higher redness (a*, 8.14) and yellowness (b*, 28.30) than instant powder obtained with ME (7.71, 24.96, *p* < 0.05). Moreover, as shown in Table 3, there was no significant difference was observed in color acceptability of two instant powder samples (*p* > 0.05), which suggested that the instant powders could be accepted by consumers. Overall, these two extrusion methods have negligible effects on color attributes from the view of consumers.

**Table 3:** The color attributes, DG and hydration characteristics of different instant powder samples.

Materials	L*	a*	b*	WI	DG (%)	WAI (g/g)	WSI (g/g)	SP
IE80	65.88 ± 0.27b	8.14 ± 0.16a	28.30 ± 0.26a	54.93 ± 0.13b	68.70 ± 0.20b	2.63 ± 0.15a	0.55 ± 0.03b	5.84 ± 0.32a
ME80	67.95 ± 0.16a	7.71 ± 0.18b	24.96 ± 0.20b	58.65 ± 0.28a	75.62 ± 0.38a	2.16 ± 0.04b	0.62 ± 0.01a	5.69 ± 0.11a

Different letters in the same column indicate significant differences (*p* < 0.05). WI, whiteness index; DG, degree of gelatinization; WAI, water absorption index; WSI, water solubility index; SP, swelling power.

### 3.3. Degree of gelatinization

The starch gelatinization process is defined as the destruction of the molecular order in the starch granules, including all accompanying and irreversible changes leading to the change of its properties. In general, gelatinization degree is negatively correlated with crystallinity (32). Previous research suggested that extrusion would result in the disruption of hydrogen bonds and crystallinity structure in starch granules, increasing the degree of gelatinization (33). The DG of different instant powder samples is shown in Table 3. The instant powder obtained with IE showed lower DG (68.70%) than instant powder obtained with ME (75.62%, *p* < 0.05), indicating that the two extrusion modes have varying effects on ordered structure of starch crystallinity. On the other hand, it has been reported that extrusion caused the formation of amylose-lipid complex and its supramolecular structure and then retained the crystallinity structure, which decreased the degree of gelatinization (15, 34). Moreover, in contrast to ME, the instant powder obtained with IE possessed a higher flavonoids content (Table 2) that can more effectively prevent the development of starch-iodine complex, decreasing the DG value (35). Therefore, the difference of DG between IE80 and ME80 could be influenced by these two factors.

### 3.4. Hydration characteristics

The WAI refers to the integrity of starch in aqueous dispersion, which depends on the availability of hydrophilic groups that bind water molecules and the gel forming ability of amylose. Reducing WAI is facilitated by the interaction of protein with amylose and amylopectin (36). The increase of WSI indicates the degradation of starch during extrusion. The SP present the swelling degree of starch after the crystallinity structure is destroyed (37). Consequently, we could evaluate the soakage of instant powder using these indicators. The WAI, WSI and SP of the two different instant powder samples are presented in Table 3. The instant powder obtained with IE showed significant higher WAI (2.63 g/g) compared to instant powder obtained with ME (2.16 g/g, *p* < 0.05). The possible reason was that the high protein content in the instant powder obtained with IE exhibited a strong ability to compete for water with other ingredients, which led to an increase in WAI (19). It has been reported that the mechanical disintegration of starch granules and polymers will be aided by the high shear force produced by extrusion, thereby increasing the value of WSI (38). The WSI (0.55 g/g) of the instant powder obtained with IE was lower in comparison with instant powder obtained with ME (0.62 g/g, *p* < 0.05), indicating that there are differences between the two extrusion modes in terms of how much starch and polymer are degraded. Moreover, heat treatment resulted in the loss of solubility of other molecules or the creation of insoluble components, masking the increase in soluble starch (38). This might contribute to the variation in WSI as well. Despite having a somewhat lower WSI than ME80, IE80 had a rather high WAI. This indicated that the instant powder obtained with IE might have better soakage property in contrast with the instant powder obtained with ME. Two extrusion modes had no significant effect on SP of instant powder (*p* > 0.05). Previous studies have shown that there were two main factors responsible for the change of SP. One was that the crystalline structure of starch was broken down during the extrusion process, and the liberated amylose and amylopectin can form hydrogen bonds with water molecules to increase SP (39). The other was that the existence of non-starch components (protein, fat, fiber, etc) in instant powder samples would reduce SP. For instance, amylose-lipid complexes have been shown to restrict SP (37).

### 3.5. α-glucosidase inhibitory activity

**Figure 3:**
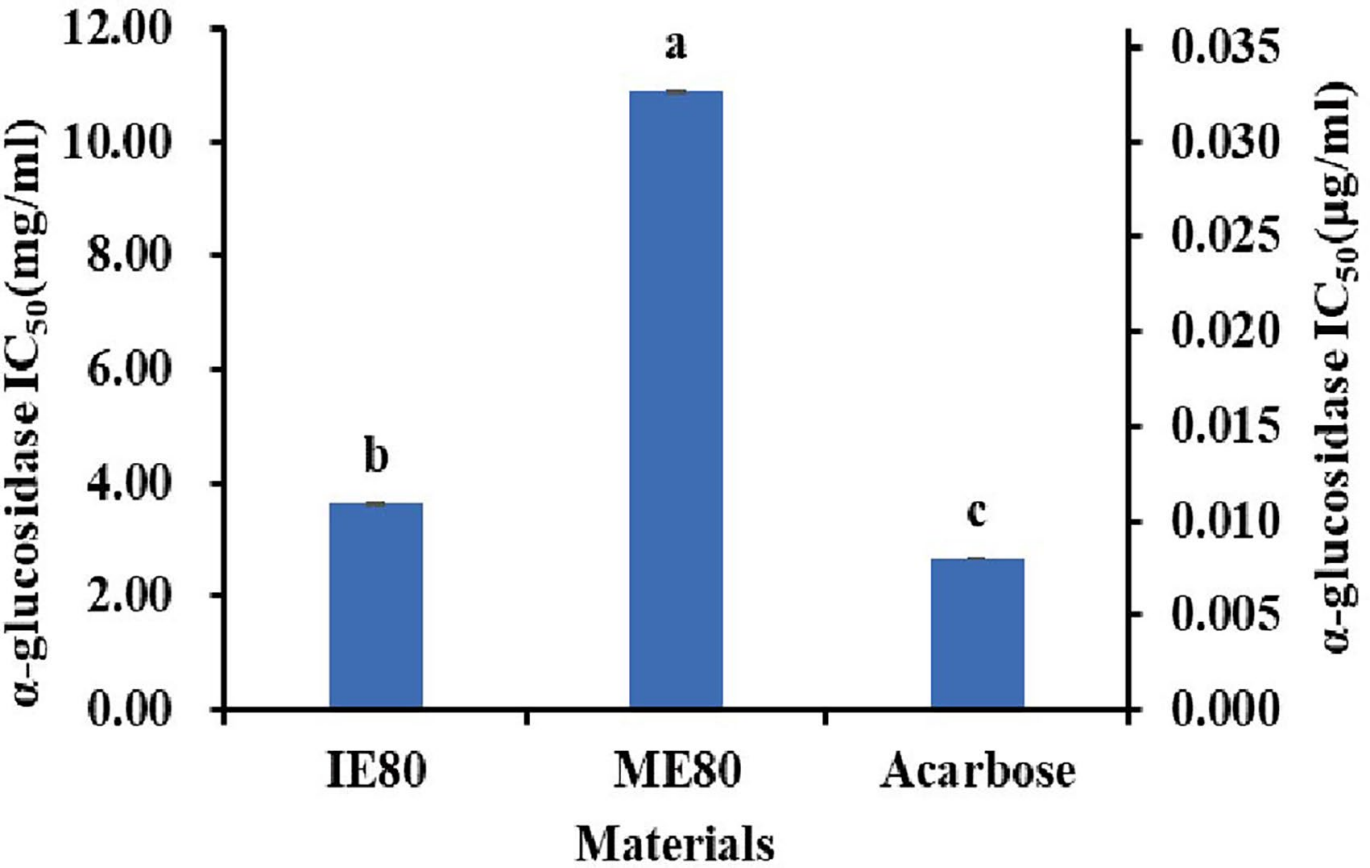
The α-glucosidase inhibitory activities of different instant powder samples. The left y-axis represents the IC_50_ scale of the sample, and the right y-axis represents the IC_50_ scale of the acarbose. Different letters (a, b, c) indicate significant differences (*p* < 0.05).

The α-glucosidase inhibitors could delay the release of glucose and the absorption of carbohydrates by inhibiting the activity of carbohydrate hydrolase in the small intestine. Therefore, natural α-glucosidase inhibitors as oral drugs have attracted great attention in patients with diabetes (40). The α-glucosidase inhibitory activity of flavonoid extract from instant powder was investigated *in vitro* (Figure 3). The instant powder obtained with IE showed significant higher inhibitory activities (IC_50_ = 3.63 mg/mL) than instant powder obtained with ME (IC_50_ = 10.90 mg/mL, *p* < 0.05), which was consistent with the trend of total flavonoids content (Table 2). Previous studies also suggested that the increase of α-glucosidase inhibitory activity is directly related to the phenolic oxidation, polymerization or changes in flavonoids profile during thermal processing (41).

### 3.6. Sensory analysis

Sensory analysis of different instant powder samples is summarized in Table 4. Overall acceptability is one of the important parameters for consumers to accept new products (29). As shown in Table 4, there was no significant difference in the scores to give overall acceptability for samples of IE80 and ME80 which were 80.75 and 81.63, respectively, indicating that these two kinds of instant powder are all acceptable to consumers. Similarly, no significant difference was observed in the taste, aroma, color, tissue state, and soakage of instant powder samples (*p* > 0.05), suggesting that both IE and ME have no significant impact on the sensory quality of these two instant powder samples.

**Table 4:** Sensory evaluation parameters of different instant powder samples.

Materials	Taste	Aroma	Color	Tissue state	Soakage	Overall acceptability
IE80	24.13 ± 1.36a	17.38 ± 1.06a	16.50 ± 1.20a	12.13 ± 1.55a	10.63 ± 0.52a	80.75 ± 2.31a
ME80	24.25 ± 1.49a	18.13 ± 1.13a	16.25 ± 1.49a	12.25 ± 0.71a	10.75 ± 0.71a	81.63 ± 2.45a

The results were expressed as means ± STD (n = 8). Values with different letters in the same column are significantly different at *p* < 0.05.

### 3.7. *In vitro* starch digestibility

**Figure 4:**
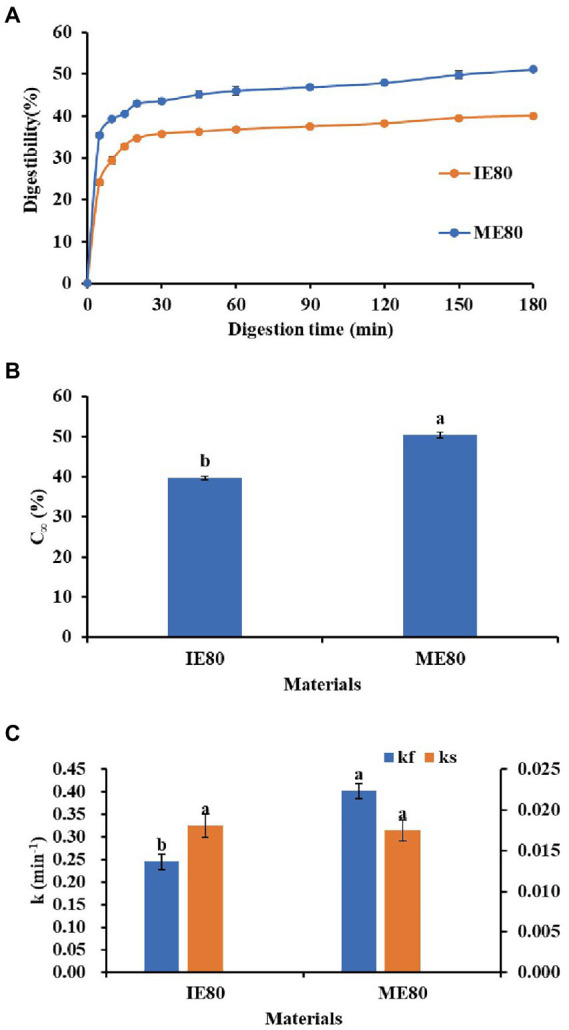
*In vitro* hydrolysis curves **(A)** and digestibility parameters C_∞_ **(B)**, k_f_ and k_s_ **(C)** from LOS plot of different instant powder samples. k_f_, starch digestion rate constants for the fast step; k_s_, starch digestion rate constants for the slow step; C_∞_, the theoretical of starch digested at the reaction end point.

The *in vitro* starch hydrolysis curves of the instant powder samples were shown in Figure 4A. The hydrolysis rate of two samples increased rapidly during the first 20 min and gradually levelled off thereafter. Recent research has shown that *in vitro* starch digestibility of Tartary buckwheat depends on food composition and microstructure formed during food processing (42). The susceptibility of two instant powder samples to the enzymatic hydrolysis was analyzed in this work. As shown in Figures 4A,B, by comparing the digestibility of starch at 180 min and the C_∞_ value, we concluded that the instant powder obtained with ME showed significantly higher digestibility compared to instant powder obtained with IE (*p* < 0.05). This was consistent with the DG trend of instant powder samples shown in Table 3, indicating that gelatinization could promote starch digestibility. Zhou et al. (42) also reported that the digestibility of Tartary buckwheat starch was greatly improved after gelatinization (42). In detail, DG is the main determinant of the starch digestion rate, while enzyme contact/combination is the primary limiting factor of starch digestion rate (43). In addition, previous studies have shown that the addition of phenolic compounds such as quercetin can not only increase the enzyme resistance of starch, but also inhibit the swelling of starch granules, thus reducing the degree of digestion (42, 44). Consequently, flavonoids are thought to be another key element causing the variation in digestibility.

### 3.8. The fitted digestion rate coefficient

**Figure 5:**
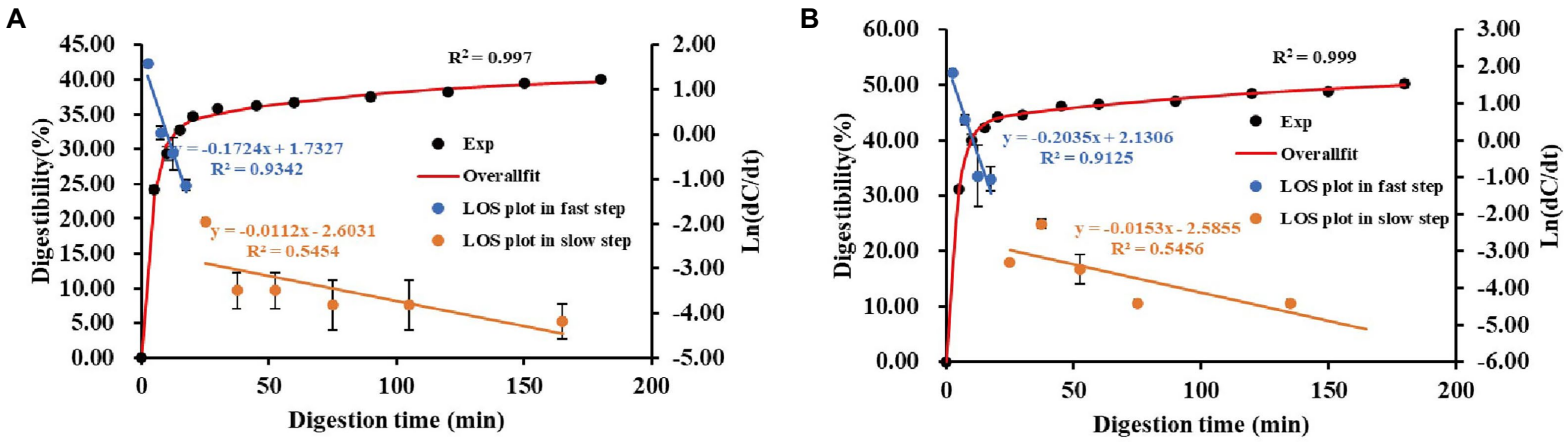
Typical starch digestion curves, overall fit curves and LOS plots from different instant powder samples. **(A)** individual extrusion; **(B)** mixing extrusion. All of the points in the LOS plots are linearly treated by least-squares fit. All the LOS plots can be divided into two parts with linear lines of different slope: k_f_ and k_s_ represent starch digestion rates for initial fast step and latter slow step, respectively. The R-squared values relate to the LOS plots and overall fit curves. The part of the LOS plot describing k_f_ is shown in blue, and the part describing k_s_ is shown in orange. Digestion data are shown in black points and overallfit curves in a red solid line.

Figure 5 shows typical experimental starch digestion curves and LOS plots for different instant powder samples. In addition, a visual comparison of k values was shown in Figure 4C. Specially, there was an initial linear step with a significantly higher rate coefficient (k_f_) and a significantly lower rate constant (k_s_) for all starchy samples, suggesting starch digestion proceeded successively with a fast and slow step. According to previous study (45), this result revealed a typical dual-stage digestion pattern for two instant powder samples. Similar with starch digestibility, the instant powder obtained with ME showed significantly higher k_f_ (0.4024) in comparison with instant powder obtained with IE (0.2456, *p* < 0.05), whereas there was no significant difference in k_s_ value (0.0181 for ME80, 0.0175 for IE 80, *p* > 0.05). This indicated that the fast-digesting component of starch in instant powder was significantly impacted by these two extrusion modes. Generally, differences in the hydrolysis kinetics of starch are attributed to the interplay of many factors, such as starch source, granule size, the degree of molecular association among chemical components among others (45). de la Hera et al. (46) demonstrated that non-starch substances alter the rate of hydrolysis by inhibiting the availability of α-amylase.

### 3.9. The starch fractions and estimated glycemic index analysis

As shown in Table 5, higher levels of RS (23.45%) and lower levels of RDS (69.31%) were observed in the instant powder obtained with IE than in ME (15.87, 75.34%, *p* < 0.05). This indicated that instant powder obtained with IE had greater resistance to the enzyme hydrolysis than instant powder obtained with ME probably due to the lower DG in IE80 (Table 3). Wang et al. (43) also reported that the susceptibility of starch from potato and lotus seed to enzymic breakdown increased with DG. Besides, the property difference between IE80 and ME80 could be due to the difference in protein, amylose, and amylopectin content and their different arrangements, and further research needs to be explored. On the other hand, the interaction between flavonoids (rutin, quercetin) and pregelatinized starch *via* hydrogen bonding should be responsible for the higher RS content in IE80 (44). Previous study have shown that the starch-polyphenol inclusion complex is resistant to digestive enzymes (47). Therefore, we hypothesized that flavonoids in Tartary buckwheat formed a complex with starch when extruded individually, whereas the formation of this complex was diluted due to the addition of adzuki bean during ME. Although there were phenolic substances in adzuki bean, they are almost degraded during extrusion. In addition, previous study reported that the starch content of adzuki bean ranged from 28.50 to 42.09% (10), which was less than 70% of starch content in Tartary buckwheat (7). Thus, the starch and phenolic substances form fewer inclusion complex during ME. Further research is needed to confirm this hypothesis.

**Table 5:** The starch fractions, HI and eGI of different instant powder samples.

Materials	RDS/%	SDS/%	RS/%	HI	eGI
IE80	69.31 ± 0.99b	7.24 ± 0.03b	23.45 ± 0.96a	58.92 ± 0.25b	72.06 ± 0.14b
ME80	75.34 ± 1.18a	8.78 ± 0.69a	15.87 ± 0.78b	74.12 ± 0.48a	80.40 ± 0.26a

Different letters in the same column indicate significant differences (*p* < 0.05). RDS, rapidly digested starch; SDS, slowly digested starch; RS, resistant starch; HI, hydrolysis index; eGI, estimated glycemic index.

To predict the glycemic response, the HI and eGI were calculated and presented in Table 5. The eGI in IE80 and ME80 were 74.12 and 80.40. The instant powder obtained with IE showed significantly lower HI (58.92) and eGI (72.06) compared to instant powder obtained with ME (74.12, 80.40, *p* < 0.05). According to the values of DG and WAI, the instant powder obtained with IE was more compact, the substances were aggregated and combined and the contact between the enzymes and starch was less, leading to lower eGI value. This observation was in agreement with the RDS changes and further demonstrated that the gelatinization promoted the digestibility of starch (Table 5).

### 3.10. Pearson’s correlations

**Figure 6:**
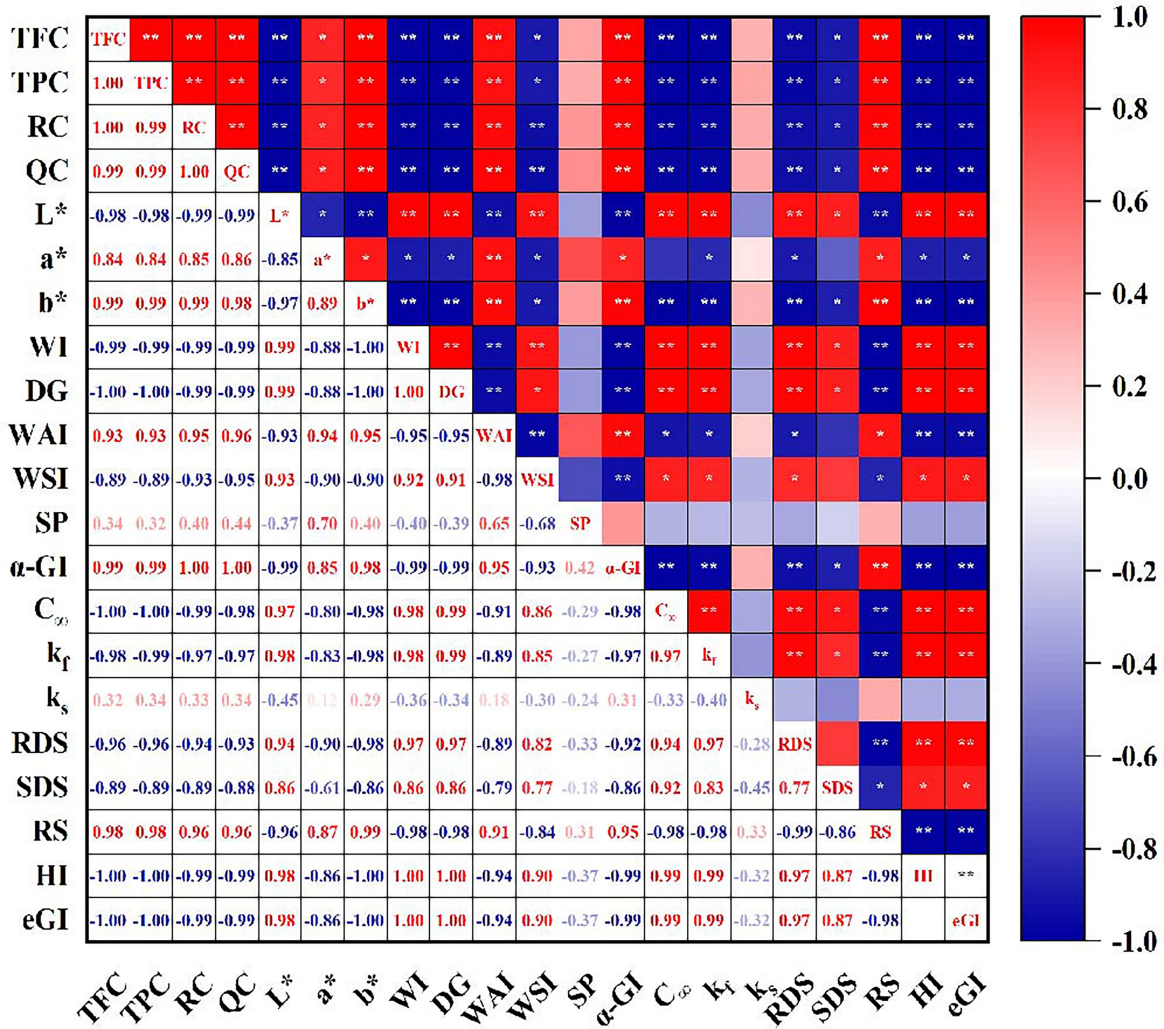
Correlation analysis among flavonoid compounds, physicochemical properties, color parameters, α-glucosidase inhibitory, digestive characteristics and estimated glycemic index. TFC, Total flavonoids content; TPC, Total phenolics content; RC, rutin content; QC, quercetin content; WI, whiteness index; DG, degree of gelatinization; WAI, water absorption index; WSI, water solubility index; SP, swelling power; C_∞_, theoretical of starch digested at the reaction end point; k_f_, starch digestion rate constants for the fast step; k_s_, starch digestion rate constants for the slow step; RDS, rapidly digested starch; SDS, slowly digested starch; RS, resistant starch; HI, hydrolysis index; eGI, estimated glycemic index. * Represents significant difference between data, *p* < 0.05; ** represents extremely significant difference between data, *p* < 0.01.

The correlation analysis among flavonoid compounds, physicochemical properties, color parameters, α-glucosidase inhibitory, digestive characteristics and estimated glycemic index are shown in Figure 6. The content of total polyphenols, total flavonoids, rutin and quercetin showed a significant correlation with L*, a*, b*, WI, DG, WAI, WSI, C_∞_, k_f_, RDS, SDS, RS, HI, eGI and α-glucosidase inhibition (*p* < 0.05). This indicated that phenolic compounds are an important factor affecting the color parameters, hydration characteristics, starch digestibility as well as α-glucosidase inhibitory activity. The α-glucosidase inhibitory activity and RS contents were significantly and positively correlated with the levels of total polyphenols, total flavonoids, rutin and quercetin as well as WAI, and significantly and negatively correlated with the DG and WSI, whereas the C_∞_, k_f_, RDS, HI and eGI showed the opposite trend. In addition, A significant correlation was shown (*r* = 0.94, 0.92, − 0.98, *p* < 0.05) between C_∞_ and RDS, SDS, and RS, and k_f_ showed significant correlation with RDS, SDS and RS (*r* = 0.97, 0.83, −0.98, *p* < 0.05). Similar results were reported by Zhang et al. (48), indicating that fractions of RDS and SDS could lead to a higher enzymatic hydrolysis rate and enzymolysis. The eGI value was remarkably correlated with k_f_, RDS, SDS and RS (*r* = 0.99, 0.97, 0.87, −0.98, *p* < 0.05). Similar result was reported that RS content in whole buckwheat noodles was inversely related to eGI (*r* = −0.983) (33). Based on these results, we can conclude that the changes in polyphenols, flavonoids, DG and hydration characteristics caused by extrusion have synergistic or antagonistic effects on the color parameters, α-glucosidase inhibitory and starch digestibility of different instant powder samples.

## 4. Conclusion

This work indicated that the two extrusion modes induce a significant effect on the phytochemical composition, DG, hydration and color properties of different instant powder samples and strongly influences its α-glucosidase inhibitory activity and *in vitro* starch digestibility. However, there was no significant difference in the sensory properties of instant powder. The instant powder obtained with IE could retain more protein and ash content as well as higher WAI, whereas the instant powder obtained with ME could retain more starch and fat content as well as higher DG. In particular, the instant powder obtained with IE showed particularly low levels of digestibility and fast digestion rate coefficient, as well as higher RS content and α-glucosidase inhibitory activity. These findings indicated that instant powder obtained with IE could serve as an ideal functional food resource with anti-diabetic potential.

## Data availability statement

The original contributions presented in the study are included in the article, further inquiries can be directed to the corresponding authors.

## Author contributions

ZZ: investigation, data curation, and writing–original draft. YL: methodology and formal analysis. LZ: material and formal analysis. YX: software and formal analysis. ML: visualization. BX, ML, and YH: writing–review and editing. GR: methodology and writing–review and editing. LZ: supervision and resources. PQ: supervision, conceptualization, and funding acquisition. All authors contributed to the article and approved the submitted version.

## Funding

This work was supported by grants from National Key R&D Program of China (Grant No. 2020YFD1001400), Shanxi Provincial Key Research & Development Project (202102140601014), and Agricultural Science and Technology Innovation Program of the Chinese Academy of Agricultural Sciences (Grant No. CAAS-ASTIP-2017-ICS).

## Conflict of interest

The authors declare that the research was conducted in the absence of any commercial or financial relationships that could be construed as a potential conflict of interest.

## Publisher’s note

All claims expressed in this article are solely those of the authors and do not necessarily represent those of their affiliated organizations, or those of the publisher, the editors and the reviewers. Any product that may be evaluated in this article, or claim that may be made by its manufacturer, is not guaranteed or endorsed by the publisher.
